# On the Treatment of Gout

**Published:** 1807-08

**Authors:** 


### *To the Editors of the Medical and Physical Journal.*

GENTLEMEN,

I Observe in your Journal of this month, an alleged case, in which the local application of a saturnine solution is stated to have been productive of very alarming consequences. The case, I think, as at present recorded, is sadly deficient in detail, and can scarcely be looked upon as a decisive example of *gastrodynia* * arising from repelled gout in the foot. The cause of complaint not only seems *incautiously* assumed; but there appears some ambiguity in the nature of the complaint itself. But admitting the correctness of the views which have been taken of this case, and two others alluded to, we must be allowed to differ widely in our sentiments from the practical application which has been drawn from them. The difficulties which occur in our science, in an especial manner, in forming a solid judgement from experience, are great and numerous; this discouraging truth, indeed, we are taught in our first aphorism. But these difficulties, your correspondent, Mr. Pope, has on this occasion avoided, by forming a precipitate and rash conclusion, on the foundation of the result of merely three ambiguous cases of gout. Mr. Pope, in a serious exhortation, advises us to condemn the cooling treatment of the disease, without any reserve, as a most obnoxious practice. These are his words, " Let us not, in this enlightened age, give up *that knowledge we have of the animal economy,* and instead of employing safe and sure means, make use of those almost approaching to *empiricism.*" But though Mr. Pope thus advises us to abandon the means of diminishing the topical inflammation in gout as empirical, he acknowledges, at the same time, that they have sometimes " *acted like a charm,*" and have " had the most instantaneous and *pleasing effects.*"

But I must observe, Mr. Pope appears in some measure, to apprehend that the three cases of gout, which he has brought forward, *may not* have the desired effect of exciting total antipathy to the remedy which was employ-

M 2 ed

* I shall make use of this term to express the complicated derangement of the stomach, which takes place in retrocedent gout.

ed in them. I cannot otherwise account for any nec essity on Mr. Pope's part, of *imagining cases* of deplorable and destructive effects being produced in "many instances," by the local inflammation in a paroxysm of gout subsiding from the application of cold. Mr. Pope tells us, he is "ready to admit, that in *many instances* cold has been productive of metastasis, producing *death*, and *very often* states nearly bordering upon it." Pray, gentlemen, how "many instances" of cold producing death, does Mr. Pope's fertile imagination choose to admit? One hundred, or one thousand? That metastasis of gout (to use an incorrect phrase) has frequently proved fatal, we have abundance of proof, and sufficiently obvious reasons—the metastasis arises from the *natural progress of the disease*, and it has been furthered instead of being arrested. The agency of *cold*, in such cases, is perfectly out of the question, and for the best of all possible reasons, it has been studiously kept aloof (I had almost said for some yards) by the interposition of *warm* rollers of flannel. The temperature of the patient's apartment, however low, can no more act upon his extremities than the climate of Nova Zembla, however severe, can at this distance act upon *ours*. Thus I am perfectly at a loss to conjecture in what manner Mr. Pope has been able to detect such disasterous consequences of the employment of cold; because this mode of treatment has but very lately been introduced in modern practice; is at this time little in vogue; here and there only a case has occurred to a practitioner; and they have been construed into objections. If then Mr. Pope *knows* of many instances of gout in which cold has had fatal effects, a reference to these instances must be of the utmost consequence; for then two opinions, I will venture to say, cannot exist upon the subject. I have thought proper to insist upon Mr. Pope's imaginary objections, in part, on this account; the question of cold, as a topical remedy in attacks of gout, has given rise to much declamatory contention; and Dr. Kinglake has been attacked, in consequence, from all quarters, and on different occasions, with explosions of eloquence; but the lack of facts we are now able to explain; they have been considered *as matters of course*, and *admitted* accordingly.

I am induced, gentlemen, to offer a few desultory remarks on the pathology and treatment of *gout*, because I am persuaded *there are forms* of this complicated disease, in which the application of cold will prove a highly beneficial remedy, and *these forms are well defined*. Further, the the discussion which Dr. Kinglake's proposal of cold, as a powerful resource for this disorder, has excited, appears to have been conducted on confined views of its history and management; so that the practice has been extolled by some and decried by others, without sufficient discrimination.

If I had any intention of going over the whole treatment of gout, of course, the management of patients, in the intervals, would be taken up at a considerable length; for it is particularly in the way of prevention that our exertions are so eminently successful; and certainly the predisposition to gout, of all others, may by the power of prophylactics, be rendered completely dormant. Bodily exercise, strict temperance, and due perseverance, we know, has on many occasions warded off the paroxysms, and extinguished all tendency to them. Hence the laconic prescription for a podagric, "Work for sixpence a day and live upon it." I will only observe further, that sea-bathing, whilst the constitution is unbroken by an irregular life, or repeated attacks of the disease, may be looked upon as a serviceable part of the regimen. We know the great connection gout has with dyspepsia, and that a little indigestible matter in the stomach is frequently sufficient to excite a paroxysm. Cold bathing is the most availing resource we possess in dyspepsia, and on this ground I recommend it, to give tone to the digestive organs, as well as to invigorate the whole system: and thus we may produce, in some measure, the double effect of diminishing predisposition upon the number of exciting causes.

It would be trifling to urge the propriety of removing a paroxysm of gout on its own account. The condition of the patient from excessive irritability and urgent pain is a sufficient motive, if his sufferings are to be held in any estimation. Before, however, stating those forms of gout, which appear to me to justify and require the topical application of cold, I may observe that the recommendation of cold as an useful and secure remedy, does not merely rest with modern advocates. So far back as the time of CELSUS, we find the employment of cold in certain forms of the gout sanctioned. *Celsus,* who is acknowledged to have been a cautious and judicious practitioner, prescribes cold as a local remedy in gout, and speaks of it with praise. When the joints of the hand or foot are affected with gout, he says, if there is tumefaction and increased heat, cold is more useful than warmth, "Seu vero tumor calorque est, *utiliora* sunt refrigerantia, RECTEQUE IN

AQUA QUAM FRIGIDISSIMA ARTICULI CONTINENTUR; sed neque quotidie, neque diu sit, ne nervi induréscant. Imponendum vero est cataplasma quod refrigeret: neque tamen in hoc ipso diu permanendum sed ad ea transeundum, quæ sic reprimunt ut emolliant. [De Re Med. lib. IV. cap. XXIV.] I consider the estimation in which Celsus held the cold treatment of the more importance, because he appears to have paid much attention to the practical uses of cold in other diseases; hence the *coincidence* is of greater value.

It is, however, undeniable that we have experience to go upon, amounting to positive proof, that the application of cold, in several cases of gout, has proved very beneficial in its consequences. Mr. Pope says that it has acted like a charm, producing the most instantaneous and pleasing effects. But in objection, cases of gout are adduced, in which the same treatment is said to have been the cause of imminent danger. Now, to avoid all unnecessary discussion, I think it may be *conceded that these cases are proofs of the pernicious effects of cold,* in particular states of the constitution, and in particular forms of the disease. But in the early periods of gout, when our resources for palliation or cure can vigorously be put in practice, the form of the disease and state of the constitution are, in a practical point of view, entirely distinct. If gout did not assume most important changes in its nature and character, during its progress, the effect of any agent upon the disease would be at all times steady and constant; and no possible reason could be assigned under these circumstances for any variation of treatment whatever. But as gout, in gaining upon the constitution, undergoes changes from one complicated form to another, a multiplicity of resources is absolutely necessary, and practice without discrimination is out of the question. In different patients, we find all the difference of age and youth; of great bodily strength, and of a broken constitution. So that this, not merely in the selection of cold, the condition of the patient, and the state of the disease, must be scrupulously regarded; for it is in this way only, that the propriety or impropriety of employing any one of our routine of remedies can be determined upon. To decide, therefore, upon the utility of a remedy, and to declare it dangerous and destructive in every stage of a disease, from its supposed effects in two or three cases, without giving the changes in its character and nature, which take place during its progress, *one thought*, is in the highest degree reprehensible.

ble. Now, it is in the early periods of gout, when the constitution is not undermined by repeated attacks, that it is our business to endeavour to diminish the severity of symptoms, and to shorten the duration of the disease by the topical application of cold. The practice, under these circumstances, seems perfectly secure. The danger of retrocession, as I before observed, arises from the natural progress of the disease, and is a *motive* to shorten its existence by every effort we can command. I appeal to the history of the disease for the proof of this position. The stomach and the extremities are the parts under consideration. We see a paroxysm of gout is connected with derangement of the stomach, and a peculiar inflammation of the joints of the extremities. The affection of the stomach is frequently in the first paroxysms altogether trifling. The paroxysms, by frequent recurrences, increase in violence and duration. The inflammation of the joints becomes more severe and extensive, and the large joints at last are affected. The derangement of the stomach increases in the number and aggravation of its symptoms; *gastrodynia* at length comes on, and *withdraws* the inflammation of the joints, on the principle of equilibrium of action, [under which we *arrange* a number of facts—See Burns, on Inflammation] and this state of the disease is called by the hypothetical and misleading phrase, Retrocedent Gout. I appeal to the history of the disease, which we know is drawn up with exquisite minuteness and accuracy, for the proof of these positions, and we there find proof, in a greater variety than we could perhaps have anticipated, that gastrodynia arises, independently of the morbid state of the joints, in so far as it *causes* the morbid state to subside. We see clearly that gastrodynia springs up, as it were, sua sponte, and that it would continue to do so merely from the progress of the disease, if the morbid state of the joints was not one of the symptoms of it. In short, that the disappearance of the local inflammation in retrocedent gout is the *effect* of gastrodynia.

This pathological view I take of Retrocedent Gout, is in consonance with the whole history of the disease. In the commencement of gout, we see it more especially produce direct effects upon the stomach; for dyspepsia is the first symptom evolved. In the progress of gout, we see its direct influence increase most considerably, pro ratâ parte, upon the stomach, till it in a manner annihilates all its functions, and then fixes upon it altogether and forms the atonic state of gout. Why then should gastrodynia dynia alone be reduced to *such extremities* as to require the agency of the great toe! Thus we find, that gastrodynia is but a consequence of the natural progress of the disease; and that a *repetition of paroxysms* MUST be the essential cause of it, because it *never* takes place in the early periods of the disease, when all other *circumstances, except the effects of repetition,* are in every respect the same at its commencement as at its termination. Thus we see, that error in pathology has been held forth to perpetuate imperfection of practice; and, that the discriminate employment of cold has been forbidden, because the nature of retrocedent gout has not been rightly considered. Why encourage the continuance of a paroxysm from the first, when every case of gout is an example before our eyes, that every paroxysm, according to its severity and duration, increases the predisposition? Hence, the progress of the disease is marked with increasing force and frequency of paroxysms? Hence they are attended with gastrodynia. Why suffer the disease to become unmanageable, and not early and unremittingly use our utmost exertions to suppress the progress of "an excruciating malady; which otherwisse grows withh our years, and curtails, or renders miserable, the latter half or third of the lives of those who are subject to it?" [ Zoonomia. ] The advocates for the employment of cold in the early periods of gout, contend for nothing more; they bear in mind, that every paroxysm increases predisposition; and see, that what is gained by mortifying restraints, submitted to for a tedious time, in the intervals may be all lost, by continuance and severity of the paroxysm; they recommend a remedy which promises something to shorten it and mitigate its symptoms. For doing so, they are reprobated with insinuating charges of practising with the instrument of death; of forgetting the knowledge of the animal œconomy; and almost of empiricism.

I fear, Gentlemen, that I have indulged in too much diffuseness; but it will be recollected, that I have adopted pathological views, in some degree peculiar to myself; and, as they appear of some practical importance, I was willing to unfold them with perspicuity if possible; the desire of being intelligible, and the want of leisure, I trust, will apologise for any superfluity or incorrectness of language.

I will just add, that the external application of cold, will prove of utility after the paroxysm is gone off, by restoring storing the tone of the parts. Ablution with cold water, has, in this way, proved serviceable to old podagrics, and is recommended in this view by Doctor Gregory, the Professor of the Practice of Physic.

It will be understood, that I consider it improper to repel the local inflammation of gout in patients who have been the victims of it for any considerable length of time. The connection between the stomach and gouty joints becomes intimate; the predisposition of the stomach becomes great, so that the slightest circumstance will excite it to diseases; the morbid change of the stomach, from repeated attacks, becomes more and more exquisite; the gout fluctuates between the stomach and the small joints. Even before gout reaches this extreme form, I apprehend it is not advisable to dispel the topical inflammation. I am, &c.

*July* 14, 1807. P. O.

P. S. In my last paper, for *Douteau* read *Pouteau.*

